# Causes and Clinical Characteristics of Small Bowel Bleeding in Northern Vietnam

**DOI:** 10.1155/2020/8884092

**Published:** 2020-11-27

**Authors:** Cong Long Nguyen, Khac Khiem Pham, Canh Hiep Nguyen, Hoang Nam Nguyen, Tran Tien Dao, Hoai Nam Nguyen, The Phuong Nguyen, Van Tuyen Pham, Tuan Thanh Nguyen, Anh Tuan Vu, Truong Khanh Vu

**Affiliations:** ^1^Department of Gastroenterology & Hepatology, Bach Mai Hospital, Hanoi, Vietnam; ^2^Pathology Center, Bach Mai Hospital, Hanoi, Vietnam; ^3^Department of Human Pathology, Kanazawa University Graduate School of Medicine, Takaramachi 13-1, Kanazawa, Japan; ^4^School of Chemical Engineering, Hanoi University of Science and Technology, Hanoi, Vietnam

## Abstract

**Aim:**

Causes, clinical features, and diagnostic approaches for small bowel (SB) bleeding were analyzed to derive recommendations in dealing with this clinical condition.

**Methods:**

We included 54 patients undergoing surgical treatment for SB bleeding, from January 2009 to December 2019. Detailed clinical data, diagnosis procedures, and causes of bleeding were collected.

**Results:**

Among 54 cases with SB bleeding, the most common causes were tumors (64.8%), followed by angiopathy (14.8%), ulcers (9.3%), diverticula (5.6%), tuberculosis (3.7%), and enteritis (1.9%). Most tumors (32/35 cases, 91.4%) and vascular lesions (8/8 cases, 100%) were located in the jejunum. The incidence of tumors was higher in the older (30/41 cases, 73.1%) than that in patients younger than 40 years of age (5/13 cases, 38.5%, *P* < 0.01). Common initial findings were melena (68.5%) and hematochezia (31.5%). The overall diagnostic yield of computed tomographic enterography (CTE) was 57.4% (31/54 cases), with the figures for tumors, vascular lesions, and inflammatory lesions being 71.4% (25/35 cases), 62.5% (5/8 cases), and 12.5% (1/8 cases), respectively. Double-balloon enteroscopy (DBE) definitively identified SB bleeding sources in 16/22 (72.7%) patients.

**Conclusion:**

Tumors, angiopathy, ulcers, and diverticula were the most common causes of SB bleeding in Northern Vietnamese population. CTE has a high detection rate for tumors in patients with SB bleeding. CTE as a triage tool may identify patients before double-balloon enteroscopy because of the high prevalence of SB tumors.

## 1. Introduction

Small bowel (SB) bleeding, a potentially life-threatening clinical condition, accounts for 5 to10% of all gastrointestinal (GI) bleeding sources [1, 2]. It could occur anywhere between the ligament of Treitz and the ileocecal valve. Bleeding from SB may be overt, presenting as visible hemorrhage (e.g., hematemesis, melena, and hematochezia), or occult, manifested by recurrent positive fecal occult blood test results with or without iron-deficiency anemia [3]. Historically, GI bleeding was referred to as being obscure (OGIB), if no source of hemorrhage was found after initial evaluations, including esophagogastroduodenoscopy (EGD), colonoscopy, and radiologic examination [4]. With advances in SB imaging, including video capsule endoscopy (VCE), device-assisted endoscopy, radiographic imaging, and angiography, a SB bleeding source can be now identified in the majority of patients (∼75%) previously diagnosed with OGIB. Recently, the American College Of Gastrointestinal (ACG) guideline has proposed the former term OGIB should be reclassified as SB bleeding [3, 5–7].

Patients with SB bleeding often require recurrent hospitalizations, multiple transfusions, and extensive evaluations. Early diagnosis of SB bleeding is challenging, particularly in settings of limited diagnostic techniques, due to its vague and nonspecific symptoms. Furthermore, various underlying etiologies can cause SB hemorrhage with their prevalence varying among age groups and regions [3, 4, 8–10]. Crohn's disease and SB tumors are more prevalent in patients younger than 40 years of age [11, 12]. Vascular lesions (∼66%) such as angioectasias are the most common etiology of SB bleeding in the Western population, followed by inflammatory lesions and SB tumors, while inflammatory lesions (∼38%) and tumors are more prevalent in Asia [13–20]. Few data on clinical manifestations and causes in a patient with SB bleeding have been reported from Vietnam, where advanced diagnostic tools for SB evaluations remain limited. In this study, we retrospectively analyzed 54 patients who were admitted to our hospital for SB bleeding and underwent surgical treatment. We detailed causes and clinical features and discussed practical diagnostic approaches for suspected SB bleeding to provide a more complete and up-to-date picture of this condition in Northern Vietnam.

## 2. Materials and Methods

### 2.1. Patient Population

We enrolled 54 patients with SB bleeding, who were admitted to Bach Mai Hospital, a tertiary national teaching hospital in Northern Vietnam, from January 2009 to December 2019. The Ethics committee of Bach Mai Hospital approved the research protocol, and this study followed standard ethical guidelines. The inclusion criteria were cases with a final diagnosis of SB bleeding and were treated with surgery. All patients underwent EGD and colonoscopy two times and computed tomographic enterography (CTE) to confirm SB bleeding diagnosis and exclude lesions of the stomach, duodenum, colon, and rectum. Additional methods such as angiography or double-balloon enteroscopy (DBE) were applied in a subset of patients. We collected the following data for each patient: age, sex, alcohol history, disease course, initial symptoms, clinical manifestations, amount of blood transfusion, complications, and time between initial presentation and diagnosis. Totally, 54 cases were eligible for further analysis.

### 2.2. Data Analysis

Data were presented as mean (range) for numerical values and frequency (percentages) for categorical variables. The *χ*^2^ test or Fisher' exact test was used to compare categorical data among groups. Statistical analysis was performed using SPSS software (version 23.0, IBM SPSS Statistics, Armonk, NY, USA). *P* value <0.05 was considered significant.

## 3. Results

### 3.1. Clinical Characteristics, Frequency of Hospitalization, and Complications

Among 54 patients in this study, 34 (63%) were male. The mean patient age was 54.3 (range, 18 to 82) years with 41 (75.9%) cases aged over 40 years. The median hemoglobin levels at initial evaluation were 78.7 (range, 25 to 128) g/dL, and 81.5% of the patients received at least one unit (350 mL) of packed erythrocytes (Table 1).

The number of patients diagnosed during their first admission was 33 cases (61.1%), 13 cases (24.1%) on their second admission, five cases (9.3%) on their third administration, two cases on their fifth, and one case on the sixth administration. The mean period of hospital stay was 13.4 days. SB bleeding presented for less than 1 month in 33 cases, 1 to12 months in 13 cases, and more than 12 months in 8 cases. The longest duration was 5 years on a patient admitted to the hospital six times because of recurrent suspected SB bleeding, finally diagnosed with a SB tumor on intraoperative enteroscopy.

### 3.2. Causes of Jejunoileal Bleeding

Tumors were the most common lesions, accounting for 35 (64.8%) cases, followed by angiopathy found in 8 (14.8%) patients. Ulcer and diverticula of the jejunoileal region were observed in 5 (9.3%) and 3 (5.6%) patients, respectively (Table 2).

There was no significant difference in the rate of various causes of SB bleeding between male and female patients. However, the significant difference in frequency of SB bleeding etiologies was found in patients younger and older than 40 years with tumors remaining the most prevalent cause in both age groups (Table 2, *P* < 0.01). Regarding anatomic locations of bleeding caused by tumors, the jejunum was the main site, accounting for 32/35 (91.4%) cases (Table 3).

**Figure 1:**
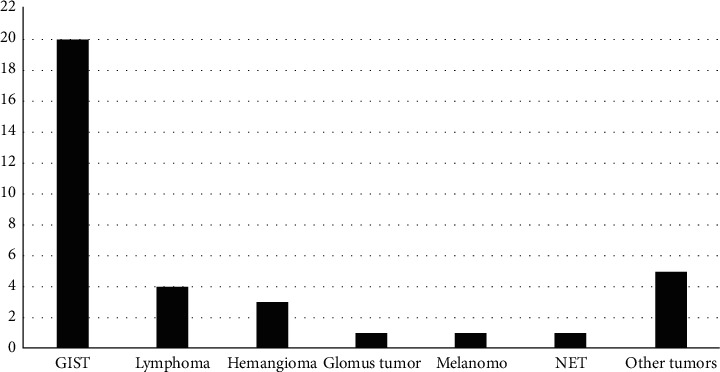
Tumor types based on the histological diagnosis. Abbreviation: GIST, gastrointestinal stromal tumor; NET, neuroendocrine tumor. Values are the number of cases.

Bleeding from the jejunum (43 cases) was more common than that from the ileum (11 cases). Besides, the frequency of each causative etiology was significantly different between the two anatomical locations. While tumors remained the most frequent causes of bleeding in both jejunum (74.4%) and Ileum (27.3%), angiopathy was found only in the jejunum (18.6%) and tuberculosis only in the ileum (Table 3, *P* < 0.01). Among tumors, gastrointestinal stromal tumors (GISTs) were the most common pathological type (20/35 cases, 57%) (Figure 1).

**Figure 2:**
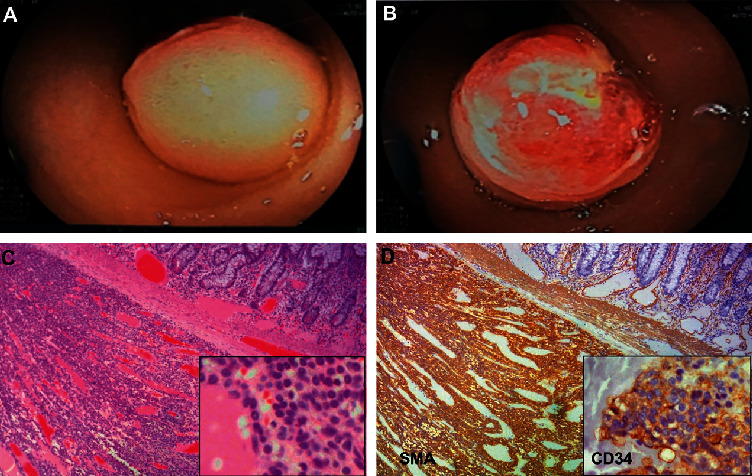
Representative images of an ileal glomus tumor. (a-b) Double-balloon enteroscopy revealed a protruded tumor with an ulcer surface. (c-d) Histological findings. (c) The tumor developed in the submucosal layer, composed of uniform glomus cells with round nuclei and amphophilic to eosinophilic cytoplasm arranging in nests, sheets, and trabeculae separated by branching vessels. Hematoxylin and eosin staining; original magnification x40 (box, x200). (d) Tumor cells were positive for SMA and CD34 (box). Immunohistochemistry staining; original magnification x40 (box, x200).

Interestingly, we found a rare case of ileal glomus tumor. The patient was 59 years old, presented with melena. A multislice computed tomography showed a hypervascular mass of 14 mm in the ileum. Double-balloon enteroscopy revealed a protruding tumor with ulceration on the surface (Figure 2).

### 3.3. Initial Symptoms and Main Clinical Manifestations

The most common initial symptom was melena, found in 37 (68.5%) patients, followed by hematochezia (31.5%) (Table 1). Abdominal pain presented in 15 patients (27.8%) in which causative lesions were 11 GI stromal tumors, two ulcers, one enteritis, and one tuberculosis (Table 2). Patients with angiopathy had no abdominal pain. The shock signs, which were defined as systolic blood pressure lower than 90 mmHg or drop of systolic pressure larger than 40 mmHg during the presentation, occurred in 15 patients (27.8%), in which 11 patients had bloody stools, two patients presented with shock signs and severe abdominal pain, one case experienced perforation caused by ileum ulcer, and one case had intussusception caused by jejunum tumor.

Regarding stool and shock signs at administration, patients with jejunal bleeding often presented with melena (74.4%) hematochezia (bloody stool, 25.6%). Those with bleeding from the ileum had melena and bloody stool in 5/11 (45.5%) and 6/11 (54.5%) cases, respectively. Patients with bleeding from the jejunum had a lower frequency of shock symptoms (25.6%) compared with those with ileal bleeding (36.4%), but the difference was not statistically significant.

### 3.4. Diagnostic Procedures

CTE evaluations were performed in all 54 cases with positive findings in 31 patients (57.4%); histological results after operations confirmed 25 tumors, 5 angiopathies, and one ulcer (Table 4).

The detection rates of CTE for SB tumor, angiopathy, and ulcer were 25/35 cases (71.4%), 5/8 cases (62.5%), and 1/4 cases (25%), respectively. Enteroscopy definitively identified the cause of bleeding in 16/22 (72.7%) patients. Of 5 patients who underwent selective arteriography, three cases (60%) were found the source of bleeding. Intraoperative endoscopy definitively identified the source of bleeding in three patients.

## 4. Discussion

Various underlying lesions can cause SB bleeding, which may alter the efficacy of diagnosis and treatment approaches. In Western countries, the causes of SB bleeding have been well established, with angioectasias being the most common (29–60.1%) [15, 16, 20–22]. In several meta-analyses, the diagnostic yields of VCE and DBE for SB vascular abnormalities were 24–58.5% and 24–41.5%, respectively [7, 23]. In this study on Vietnamese patients, tumors (64.8%) were the most common causes of SB bleeding, followed by angiopathy (14.8%), ulcer (9.3%), diverticular (5.6%), tuberculosis (3.7%), and enteritis (1.9%). Age has been recognized as a determinant of pathological characteristics of SB lesions. Patients under the age of 40 years are more likely to have inflammatory bowel disease or Meckel's diverticulum [10, 14, 24]. In contrast, we found tumors remained the leading cause of SB bleeding both in patients aged older and younger than 40 years. The ratio of SB hemorrhage caused by tumors was more frequent in the older (73.1%) than that in the younger (under 40 years) age group (38.5%; *P* < 0.01). Several previous studies reported a similar trend in the distribution of bleeding SB lesions with tumors accounted for 28.2–30% [14, 25]. Our results, however, are divergent from those of previous studies from Asia, which showed inflammatory lesions were the most common etiologies, followed by vascular diseases or tumors [11, 13, 17, 26, 27]. The divergences are possibly due to differences in patient selection among studies as well as our sample selection bias. Our cohort was included only SB bleeding cases underwent surgical intervention. However, treatment indications depend on the patient's status as well as underlying causes. Those with bleeding caused by SB tumors are more likely to undergo surgical treatment. Indeed, Green et al. reported among patients who underwent resections for SB bleeding, cases with tumors accounted for 40.2% (45/112 cases), followed by angioectasia (14.3%) [28]. Meanwhile, conservative management is the main approach for nonneoplastic lesions, such as endoscopic therapy for angioectasias, and medical management for inflammatory lesions [3, 9].

Heterogeneous tumor types, both malignant and benign neoplasms, may originate from the small intestine, but benign tumors have been found more common than the malignant among causes of SB bleeding [24, 29–31]. Benign lesions of the SB include adenomas, leiomyomas, fibromas, and lipomas, in which tumors that most frequently cause small intestinal bleeding are leiomyomas. Malignant tumors include adenocarcinomas, neuroendocrine tumors (carcinoids), GISTs, and lymphomas. In our study, most tumors were malignant types with GISTs accounting for 20/35 (57.1%). Some other rare SB bleeding etiologies in our series were glomus tumor (one case, Figure 2), representing 1–4.5% of soft tissue tumors [32], and small intestinal tuberculosis (2 cases). Massive GI bleeding is considered a rare symptom of intestinal tuberculosis [33–35]. The majority of reported cases had bleeding from the ileocecal area [36, 37].

Clinically, intermittent melena (68.5%) was the most common initial symptom among our patients with small intestinal bleeding, followed by hematochezia (31.5%) and abdominal pain. Shock signs were found in 27.8% of cases. Regarding the anatomic distribution of SB bleeding sources, our data show that most lesions (79.6%, 43/54 cases) were located in the jejunum with 74.4% of cases being tumors. Interestingly, vascular lesions were responsible for 18.6% of jejunal bleeding but not found in the ileum (Table 3). Similarly, tumors (77.8%) or angioectasias have been reported to mainly locate in the jejunum or proximal small intestine within the reach of push enteroscopy [20, 38–41]. These findings suggest repeat push enteroscopy may be more beneficial than standard EGD in patients with suspected SB bleeding. It is, however, worth noting that angioectasias are often multiple with 57–60% of patients having more than one affected location [20, 38–40].

Evaluation of suspected SB bleeding remains a great challenge for gastroenterologists. In our series, we adhere to the current recommendations of the ACG guideline for diagnosis and management of bleeding from the small intestine [3]. For patients with stable bleeding, the initial approach should be considered a second look. Upper and lower endoscopy should be performed in cases of recurrent hematemesis, melena, or a previously incomplete exam. All patients in this study underwent a second look. Recent advances in diagnostic modalities, including VCE, deep enteroscopy (DBE, SB enteroscopy, and spiral enteroscopy), and radiologic modalities, including CTE, magnetic resonance enterography (MRE), and selective angiography, have led to significant improvement in diagnosis and management of SB bleeding [29, 42, 43]. Most recent guidelines recommend small bowel VCE as the first-line procedure for SB evaluation [3, 5, 44]. However, VCE was not available at our hospital, patients were mainly diagnosed based on CTE evaluation with a detection rate of 57.4%. In prior studies, the pooled overall diagnostic yield of CTE for SB bleeding was 40% [45], which was lower than that of VCE (53–62%) and DBE (56–68%), mainly due to the low detection rate of CTE for vascular and inflammatory lesions. For SB tumor detection, CTE offers a comparable diagnostic yield to that of DBE and VCE [19, 46, 47]. Submucosal SB masses are better revealed by CTE, which may be missed on VCE evaluation. VCE and CTE, therefore, complement each other in detection of SB bleeding sources [10, 41, 48]. Our data show that CTE was able to detect 25/35 (71.4%) tumors and 6/19 (31.6%) nontumor lesions. Similarly, some other studies showed that multidetector computed tomography (MDCT) had a higher diagnostic yield for tumors compared with that for nontumor etiologies of SB bleeding (67.4–100% vs. 16.7–33.3%) [48–51]. A greater overall diagnostic yield of CTE (57.4%) in the present study could be attributed to the high prevalence of SB tumors (64.8%). These results support using CTE as initial evaluation in patients with suspected SB tumor. Also, multiphase CTE has been found useful in detecting nontumor causes of SB bleeding, including vascular lesions [50, 52, 53]. Localization of SB lesion on CTE or multiphase CT scan allows a better selection of DBE insertion route [49]. Predictors of a greater diagnostic yield for CTE in SB bleeding patients include overt bleeding, under 40 years of age, and a history of massive bleeding [8, 48, 50]. However, negative CTE findings do not exclude SB bleeding sources. Heo et al. reported a VCE diagnostic yield of 57% in suspected SB bleeding patients, following negative CTE results [54]. In this study, besides CTE, enteroscopy was used to identify bleeding lesions in 16/22 (72.7%) cases and selective arteriography in 3/5 (60%) patients. One case with a small tumor was finally diagnosed by intraoperative endoscopy because of recurrent bleeding, failed to diagnose by the other investigations. Bleeding sources were successfully identified at the first-time hospital administration in 61.1% of patients, and 38.9% were diagnosed after more than second administration. All our 54 patients underwent surgical treatment because of bleeding from SB tumors or other etiologies failed to control by conservative medical approaches. Therefore, selection of diagnostic modalities in patients with suspected SB bleeding should be individualized and depends on patient presentation and suspicious underlying causes [3].

Our study has several limitations. This was a retrospective, single-center study with an obvious selection bias, including only patients with surgical treatment. Therefore, the incidence of SB bleeding etiologies reported in our study does not exactly reflect that of the general population. Due to retrospective nature, patients did not follow the same standardized diagnostic and management protocol. Furthermore, radiologists and endoscopists, who interpreted imaging data with varying experience, were not blinded to clinical information, which may have affected our results.

In conclusion, tumors, angiopathy, ulcers, and diverticular diseases were the most common causes of SB bleeding in Vietnamese population. Most tumors and vascular lesions were located in the jejunum. Main clinical manifestations were melena and hematochezia, followed by abdominal pain and shock signs. This study suggests CTE as a triage tool may identify patients who will benefit from DBE and aid endoscopists in choosing the most efficient route.

## Figures and Tables

**Table 1:** Clinical features of patients with small bowel bleeding.

Characteristics	Values
Sex (male/female)	34/20
Age (years)	54.3 ± 17.7
Melena	37 (68.5)
Hematochezia	17 (31.5)
Abdominal pain	15 (27.8)
Shock at presentation	15 (27.8)
Amount of blood transfusion (unit)	4.9 (0–27)
Hemoglobin value (g/dL)	78.7 (25–128)
Transfusion need	44 (81.5)
Hospital stay (day)	13.4 ± 8.1
*Comorbidities*	
Hypertension	4 (7.4)
Diabetes	2 (3.7)
Liver cirrhosis	2 (3.7)
Chronic hepatitis B	2 (3.7)
Alcohol abuse	2 (3.7)
Valvular heart disease	1 (1.9)
COPD	1 (1.9)
NSAIDs	1 (1.9)

COPD: chronic obstructive pulmonary disease; NSAIDs: nonsteroidal anti-inflammatory drugs; one unit of blood: 350 mL blood. Values are *n* (%) or mean ± standard deviation (SD) unless otherwise specified.

**Table 2:** Causes and associated factors of small bowel bleeding.

Causes	Sex			Age group			Abdominal pain
	Male	Female	*P* value	≤40	>40	*P* value	
Tumor	19 (55.9)	16 (80)	0.73	5 (38.5)	30 (73.1)	**<0.01**	11
Angiopathy	5 (14.7)	3 (15)	0.72	3 (23.1)	5 (12.1)	0.72	0
Ulcer	4 (11.8)	1 (5)	0.37	1 (7.7)	4 (1)	0.37	2
Diverticula	3 (8.8)		0.25	3 (23.1)	0 (0)	0.25	0
Tuberculosis	2 (5.9)	0 (0)	0.5	1 (7.7)	1 (2.4)	1.00	1
Enteritis	1 (2.9)	0 (0)	1.00	0 (0)	1 (2.4)	1.00	1

Values are *n* (%).

**Table 3:** Correlation between anatomic locations and causes of small bowel hemorrhage.

Causes	Location, *n* (%)		*P* value
	Jejunum	Ileum	
Tumor	32 (74.4)	3 (27.3)	**<0.01**
Angiopathy	8 (18.6)	0 (0)	**<0.01**
Ulcer	2 (4.7)	3 (27.3)	1.00
Diverticula	1 (2.3)	2 (18.2)	1.00
Tuberculosis	0 (0)	2 (18.2)	0.5
Enteritis	0 (0)	1 (9.1).	1.00

**Table 4:** Bleeding causes diagnosed by various diagnostic procedures.

Diagnostic procedures	Causes of small bowel bleeding					
	Tumor	Angiopathy	Ulcer	Enteritis	Diverticulum	Tuberculosis
CTE	25	5	1	0	0	0
Enteroscopy	8	3	2	0	1	2
Selective arteriography	2	1	0	0	0	0
Intraoperative endoscopy	1	0	2	0	0	0

CTE, computed tomographic enterography.

## Data Availability

The datasets generated and analyzed during the present study are available from the corresponding author on reasonable request.
